# The Influence of Empagliflozin on the Expression of Mitochondrial Regulatory Proteins in Human Myocardium in an Ex Vivo Model of Short-Term Atrial Tachypacing

**DOI:** 10.3390/ijms26041664

**Published:** 2025-02-15

**Authors:** Paweł Muszyński, Magdalena Cieślińska, Magdalena Dziemidowicz, Elżbieta Bonda-Ostaszewska, Tomasz Hirnle, Tomasz Andrzej Bonda

**Affiliations:** 1 Department of General and Experimental Pathology, Medical University of Bialystok, Mickiewicza 2C, 15-222 Bialystok, Poland; pawel.muszynski@umb.edu.pl (P.M.); magdalena.sokolowska@umb.edu.pl (M.C.); magdalena.dziemidowicz@umb.edu.pl (M.D.); 2 Institute of Biology, University of Bialystok, Ciołkowskiego 1J, 15-245 Bialystok, Poland; elabonda@uwb.edu.pl; 3 Department of Cardiosurgery, Medical University of Bialystok, Sklodowskiej-Curie 24A, 15-276 Bialystok, Poland; kardiochirurgia@umb.edu.pl

**Keywords:** SGLT-2, mitochondrial function, mitochondrial biogenesis, atrial fibrillation

## Abstract

Atrial fibrillation (AF) is associated with energetic deficiency and oxidative stress due to mitochondrial dysfunction, resulting in electric remodeling. Long-term treatment was found to ameliorate mitochondrial function and decrease inducibility in animal models. No studies examine the short-term effect of SGLT-2 inhibitors administration in AF. In the present study, the samples of the right atrial appendage collected from 10 patients subjected to elective cardiac surgery were sliced and incubated in a control buffer (EMPA 0), 0.2 µmol/L empagliflozin (EMPA 0.2), or 1.0 µmol/L (EMPA 1). The expression of mitochondrial biogenesis, fission, and fusion proteins was measured by Western blot after 30 min of electrical stimulation (control—1 Hz or tachypacing—5 Hz). The PGC-1α protein expression was increased after 30 min of stimulation with 1 Hz when incubated under a higher concentration of empagliflozin. After tachypacing, EMPA 0.2 increased PGC-1α, while EMPA 1.0 upregulated NRF-1. Both concentrations increased NRF-2 during control stimulation. The oxygen consumption was higher in AF, and was decreased by SGLT-2i. Empagliflozin exerts dynamic effects on the expression of PGC-1α and other proteins involved in mitochondrial function and oxidative stress in cardiomyocytes and may modulate cellular response to tachycardia.

## 1. Introduction

Atrial fibrillation (AF) is one of the most significant problems in public health and is connected to a constantly growing burden on the healthcare system [1]. AF with a reported incidence of around 0.21–0.41 per 1000 person/years [2] is associated with a 25% mortality during the first year after diagnosis and increasing to even 42.7% within five years [1]. The importance of this disease led to the long-standing investigations devoted to its pathogenesis, complications, and management [3].

The primary electrophysiological mechanism underlying AF is re-entry, facilitated by electrical and structural remodeling of the atrial myocardium. This remodeling is characterized by slowed conduction and shortened refractoriness, resulting from ion current disturbances and cardiac fibrosis [4,5]. Impaired intracellular calcium handling leads to sarcoplasmic reticulum (SR) calcium overload and excessive reactive oxygen species (ROS) formation, promoting early (EAD) and delayed afterdepolarizations (DAD), which serve as AF triggers [4,5].

Recent clinical trials indicate that sodium-glucose cotransporter-2(SGLT-2i) inhibitors reduce AF prevalence and complications in patients with diabetes mellitus (DM) and/or heart failure [6,7,8]. However, the precise mechanism by which SGLT-2i prevent AF remains unclear. These inhibitors are hypothesized to enhance cellular energetics, mitigate oxidative stress, and improve mitochondrial function, primarily by stimulating mitochondrial biogenesis [9,10,11]. The beneficial outcomes may result from hemodynamic improvements related to volume unloading, enhanced ventricular performance, diminished atrial dilatation, or direct pharmacological effects on the atrial myocardium. This hypothesis was first proposed by Shao et al., who demonstrated that empagliflozin improves mitochondrial biogenesis, reduces atrial remodeling, and decreases AF inducibility by 48.2% in diabetic rats [12].

Proteins involved in the regulation of mitochondrial functions are dynamically modulated. Some of them exhibit expression changes within several minutes of stimulation. For instance, rapid upregulation of PGC-1α in vivo was shown in skeletal muscles after high-intensity exercise [13]. Rapid activation and intracellular translocation of this protein promote mitochondrial gene expression and boost mitochondrial biogenesis [14].

This study aimed to assess the acute effects of empagliflozin on the expression of mitochondrial function and biogenesis-related proteins using an ex vivo human atrial tachypacing model.

## 2. Results

### 2.1. The Study Population

The study population consisted of 10 patients, with an average age of 62.3 years, predominantly male (80%), with hypertension (80%) and valvular disease (80%), who were included in the study. Only patients with mitral valve stenosis were initially excluded. A total of 14 performed experiments, resulting in 92 samples for further analysis. The detailed study population characteristic is presented in Table 1.

### 2.2. Proteins Expression by Western Blot

**Figure 1:**
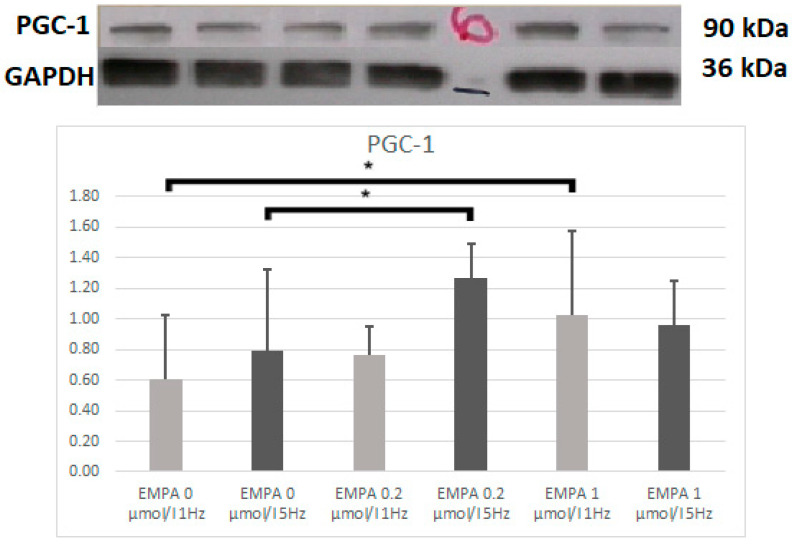
Expression level of PGC-1α according to Western blot. * *p* < 0.05 EMPA 1 µmol/L 1 Hz compared with EMPA 0 µmol/L.

The PGC-1α was higher in the EMPA 1—1 Hz than in EMPA 0—1 Hz (*p* = 0.013), with a similar tendency in EMPA 0.2 at 1 Hz. After tachypacing, PGC-1α was higher in EMPA 0.2 than EMPA 0 (*p* = 0.035) (Figure 1).

**Figure 2:**
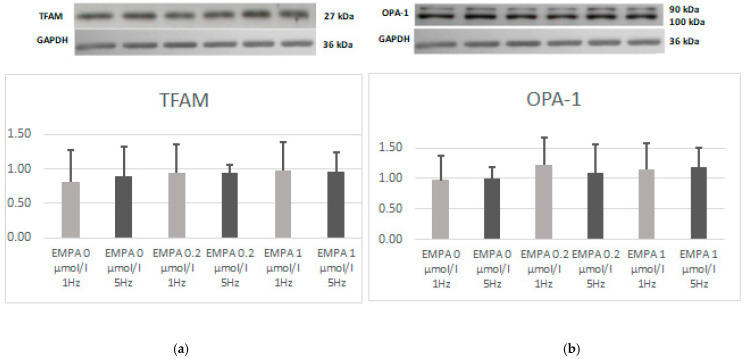
Expression level of TFAM (**a**) and OPA-1 (**b**) according to Western blot.

There was no significant difference in TFAM and OPA-1 expression (Figure 2).

**Figure 3:**
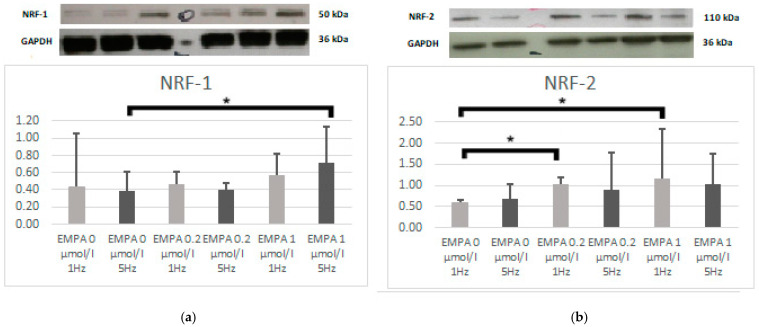
The expression level of NRF-1 (**a**) and NRF-2 (**b**) according to Western blot. * *p* < 0.05 EMPA 1 µmol/L 1 Hz compared with EMPA 0 µmol/L.

The empagliflozin in the concentration of 1.0 µmol/L significantly increased the expression of NRF-1 during tachypacing (5 Hz) in comparison to slow pacing (*p* = 0.023). The EMPA 0.2 (*p* = 0.005) and EMPA 1 (*p* = 0.008) had a higher expression of NRF-2 during slow pacing (Figure 3).

**Figure 4:**
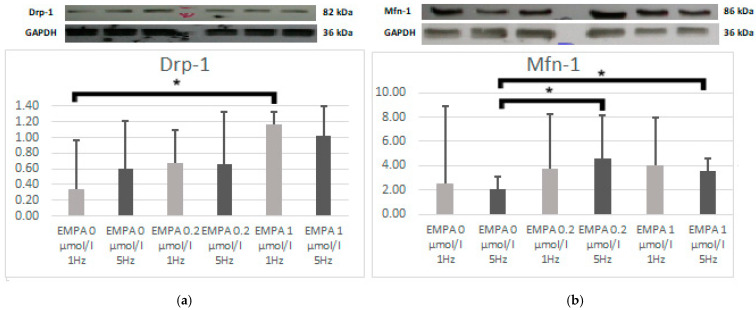
The expression level of Drp-1 (**a**) and Mfn-1 (**b**) according to Western blot. * *p* < 0.05 EMPA 1 µmol/L 1 Hz compared with EMPA 0 µmol/L.

In EMPA 1 µmol/L, the Drp-1 expression was higher than EMPA 0 during slow pacing (*p* = 0.023). There was a similar trend during tachypacing (*p* = 0.054). Mfn-1 was higher than EMPA 0 in both concentrations of empagliflozin during tachypacing (EMPA 1 with *p* = 0.036; EMPA 0.2 with *p* = 0.008) (Figure 4).

**Figure 5:**
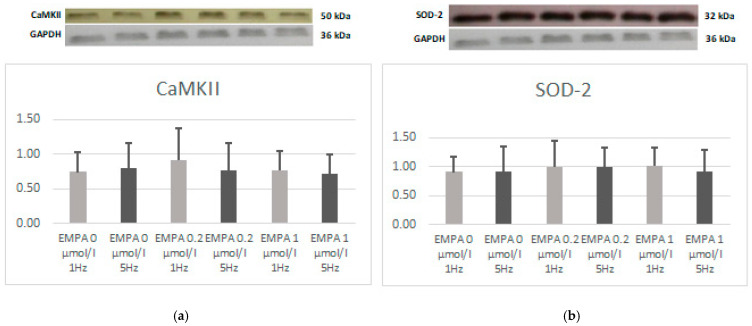
The expression level of CaMKII (**a**) and SOD-2 (**b**) according to Western blot.

**Figure 6:**
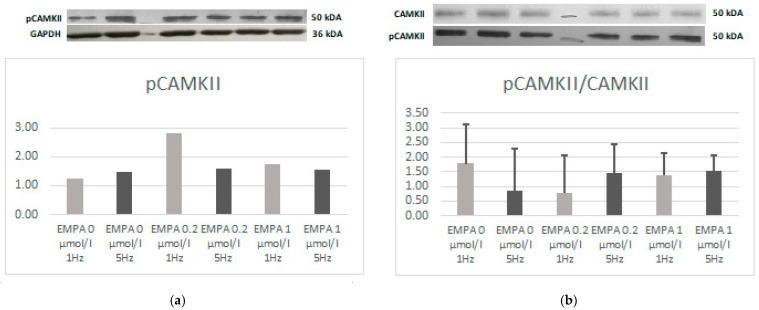
Expression level of pCAMKII (**a**) and relation of pCAMKII to CAMKII (**b**) according to Western blot.

There was no difference in the expression of CaMKII and SOD-2. There was a trend towards a higher expression of CaMKII in the EMPA 0.2 tachypacing group (Figure 5) and pCaMKII in EMPA 0.2 during slow pacing (Figure 6). The relations of pCaMKII to CAMKII seem to be lower during tachypacing in control and during slow pacing with EMPA 0.2 (Figure 6).

**Figure 7:**
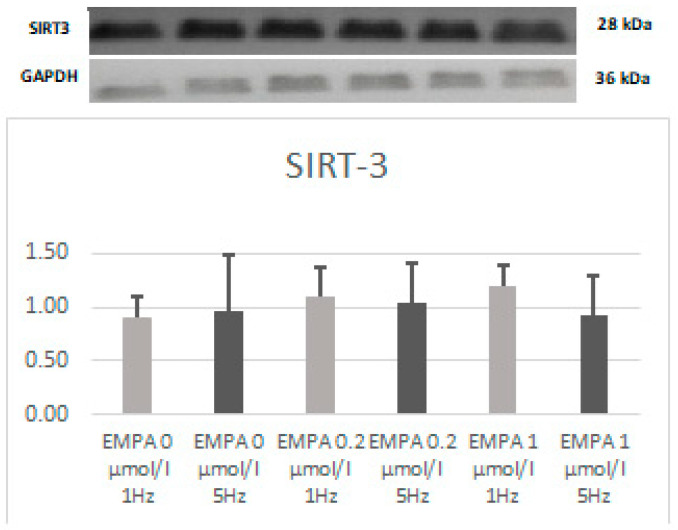
Expression level of SIRT3 according to Western blot.

There are trends toward a higher expression of 4-HNE in EMPA 0.2 (*p* > 0.05). In EMPA 1, there tends to be a higher expression of 4-HNE (*p* > 0.05). There was no difference in expression of SIRT (Figure 7).

**Figure 8:**
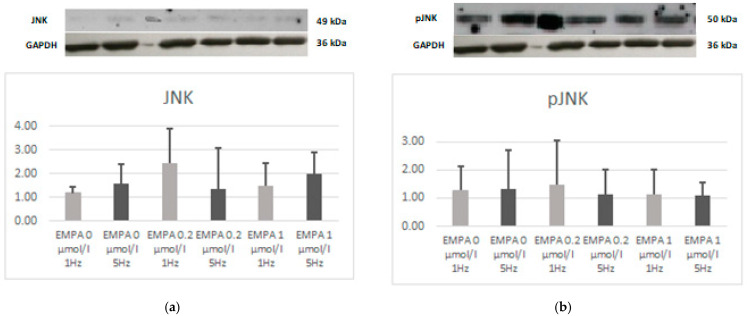
Expression level of JNK (**a**) and relation of pJNK (**b**) according to Western blot.

**Figure 9:**
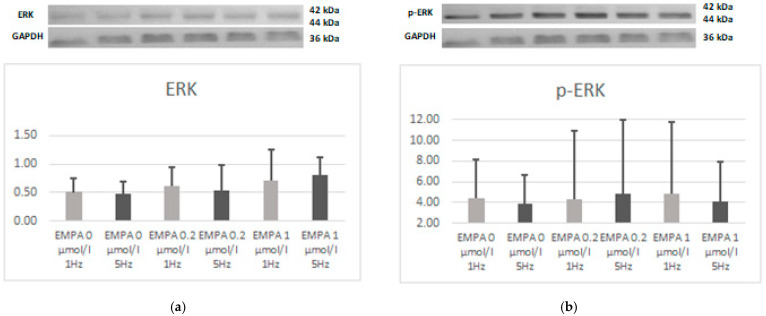
Expression level of ERK (**a**) and relation of pERK (**b**) according to Western blot.

**Figure 10:**
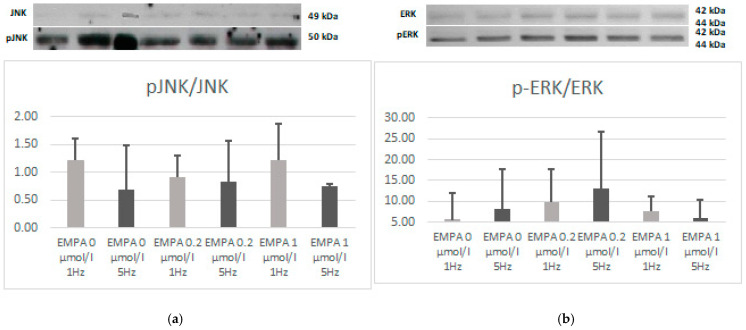
Relation of pJNK to JNK (**a**) and pERK do ERK (**b**) according to Western blot.

There was no difference in the expression of JNK, phospho-JNK, ERK, or phospho-ERK. However, the relation of phospho-JNK to JNK also tends to be lower in EMPA 0.2 1 Hz when compared to EMPA 0 1 Hz (*p* = 0.08). This can be linked to a higher expression of JNK, which also tends to be noticed in EMPA 0.2 1 Hz (*p* = 0.08) (Figure 8, Figure 9 and Figure 10).

During slow pacing in the control solution, there was a statistically significant correlation between Drp-1 and Mfn-1. When exposed to tachypacing, there was a correlation between PGC-1α and CaMKII, and between CAMKII and phospho-ERK/ERK. When exposed to empagliflozin during slow pacing, there was a positive correlation between TFAM and SOD-2. Additionally, in EMPA 0.2—1 Hz, there was a correlation between OPA-1 and CAMKII. However, during tachypacing (EMPA 0.2—5 HZ), there was a correlation between TFAM and phospho-ERK/ERK, and a negative correlation between NRF-2 and Drp-1. There was no correlation between analyzed proteins in EMPA 1—5 Hz (Table 2).

### 2.3. Oxygraphy

**Figure 11:**
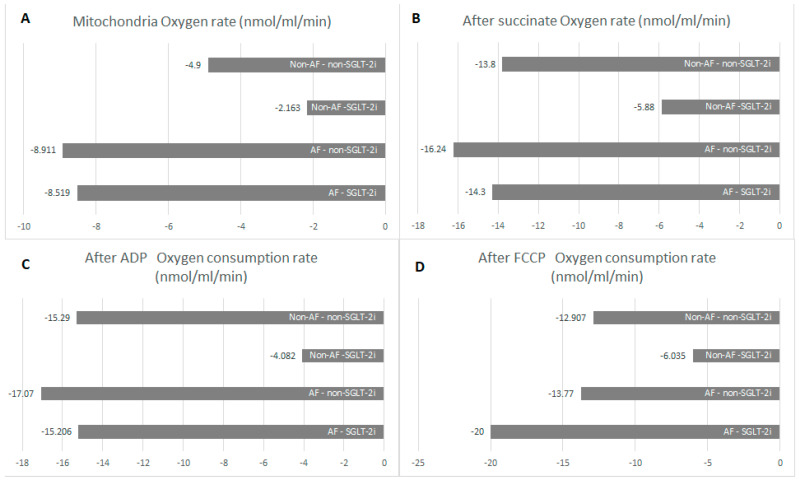
Oxygen rate after administration of atrial mitochondria (**A**), after administration of succinate (state 3, complex I  +  II, **B**), after administration of ADP (state 3, complex I, **C**), after administration of FCCP (state 3 and state 4, **D**).

**Figure 12:**
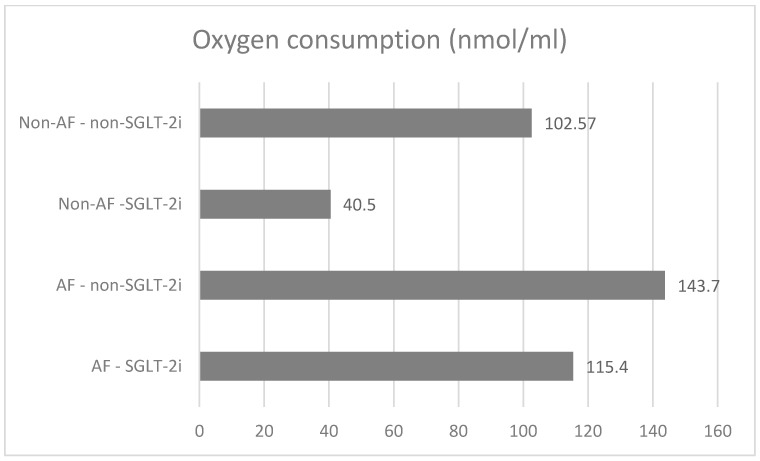
Comparison of the oxygen consumption.

**Figure 13:**
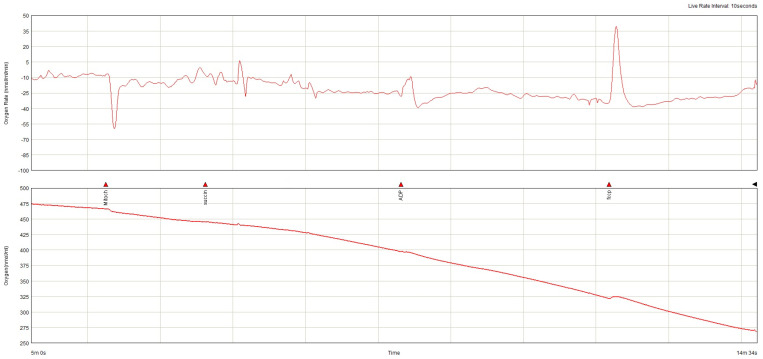
Oxygraphy (oxygen consumption rate and oxygen concentration) in ex vivo atrial mitochondria from subjects with AF treated with SGLT-2i.

**Figure 14:**
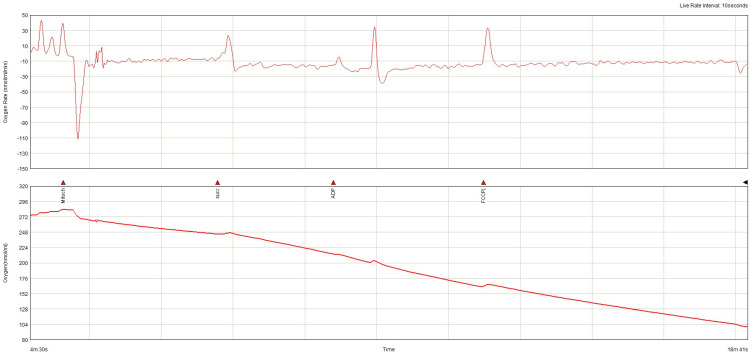
Oxygraphy (oxygen consumption rate and oxygen concentration) in ex vivo atrial mitochondria from subjects with AF not treated with SGLT-2i.

**Figure 15:**
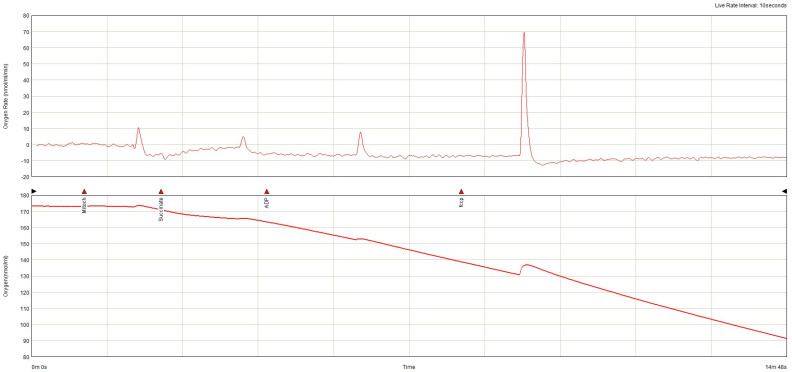
Oxygraphy (oxygen consumption rate and oxygen concentration) in ex vivo atrial mitochondria from subjects without atrial fibrillation not treated with SGLT-2i.

**Figure 16:**
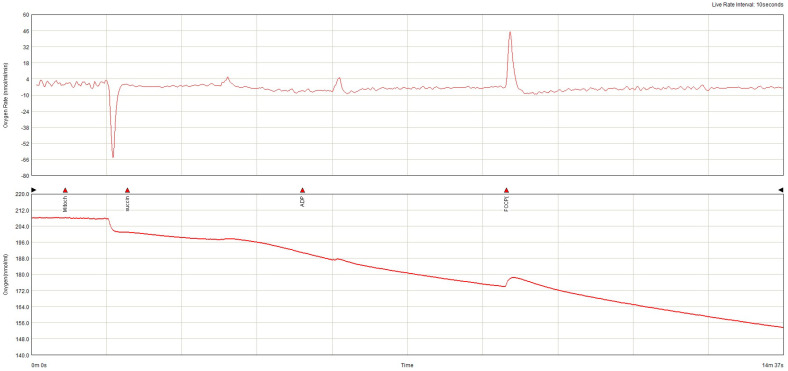
Oxygraphy (oxygen consumption rate and oxygen concentration) in ex vivo atrial mitochondria from subjects without atrial fibrillation treated with SGLT-2i.

The lowest baseline oxygen consumption rate (OCR) was found in the non-AF SGLT-2i group. Both in the absence and in the presence of AF, SGLT-2i decreased oxygen consumption. SGLT-2i in AF decreased the oxygen consumption rate during state 3 respiration, complex I  +  II, but only slightly in state 3, complex, and increased after uncoupling (Figure 11). SGLT-2i decreased OCG in all conditions in the absence of AF. The total oxygen consumption was the highest in AF without SGLT-2i, but in both conditions, SGLT-2 led to a decrease (Figure 12). The examples of oxygraphy in different settings are presented in Figure 13, Figure 14, Figure 15 and Figure 16.

## 3. Discussion

### 3.1. The Rationale for Investigating the Mechanism Behind the Effects of SGLT-2 Inhibitors on Atrial Fibrillation

AF is a relatively common comorbidity in heart failure. Evidence suggesting the beneficial effect of SGLT-2i on the incidence of atrial fibrillation and the prognosis comes from clinical trials on heart failure [6,7,8].

In EMPA-REG OUTCOME, the reduction in CV death or HF hospitalization was higher among patients with a history of AF (42% vs. 33% reduction). However, in this trial, the incidence of new-onset AF was low in both groups, with a trend toward a higher value among subjects treated with empagliflozin (1.6% placebo vs. 2.3% empagliflozin) [16].

Contrary results were observed in EMPEROR-Reduced, which focused on patients with heart failure with reduced ejection fraction. In this study, the incidence of AF decreased by 51% [16]. A meta-analysis suggests that the whole group of SGLT-2 inhibitors reduces the risk of AF development or AF/AFL-related events [6,16,17,18]. In DECLARE-TIMI58 trial, dapagliflozin reduced the risk of a first AF/AFL episode by 19% in patients with type 2 diabetes mellites (DM2) regardless of other cardiovascular comorbidities such as atherosclerosis or heart failure [6,16].

The most precise data should come from the Empagliflozin and Atrial Fibrillation Treatment (EMPA-AF) trial, which began in 2020 and investigates the efficacy of empagliflozin in reducing AF burden in patients with DM2 or overweight-associated heart failure and atrial fibrillation for whom a rhythm control strategy is indicated. In addition to the classically measured outcomes, this clinical trial also assesses quality of life [19].

Our study found that empagliflozin increased the expression of the master regulator of mitochondrial biogenesis PGC-1α after 30 min. Furthermore, we found that during tachypacing, 0.2 µmol/L empagliflozin increased the expression of PGC-1α. A similar increase was observed for Mnf-1 after treatment with both empagliflozin concentrations and for NRF-1 at a higher dose of empagliflozin.

### 3.2. Impact of SGLT-2 Inhibitors on the Pathomechanisms Involved in Atrial Fibrillation

Shao et al. showed favorable effects of empagliflozin in ameliorating arrhythmic substrate, improving electrophysiological abnormalities, and reducing AF inducibility in rats with DM. The protective effects of SGLT-2 inhibition against AF in this experimental work were attributed to the upregulation of proteins involved in mitochondrial biogenesis and improved LA tissue mitochondrial respiration [12]. Other studies also provided data regarding the influence of SGLT2i on mitochondrial homeostasis in the myocardium. In mice, 7 days of empagliflozin treatment increased mitochondrial mass, as estimated by citrate synthase activity [20]. Additionally, SGLT-2i can influence the mitochondrial fusion/fission proteins, such as Mitofusin-1 and Mitofusin-2, which may have potential in the treatment of cardiomyopathies and AF [21,22,23].

Most studies investigated longer exposure to SGLT-2i, ranging from 7 days to 8 weeks or more.

In the rat model, 7 days of empagliflozin administration did not change cardiac conduction activity. However, it decreased myocardial vulnerability to sudden cardiac death (from 69.2% to 0% in the empagliflozin group) [20]. Empagliflozin also increased the phosphorylation of cardiac ERK1/2 after reperfusion injury, and its inhibition abolished this protective effect on sudden cardiac death [20].

Significant effects of SGLT-2i were observed even after much shorter treatment periods. For example, 72 h exposure directly affected the phenotype and function of human cardiac myofibroblast, where reduced activity and cell-mediated collagen remodeling were observed [24].

Functional changes were observed even after very short treatment with SGLT-2i. In Langendorff-perfused rabbit hearts subjected to 30 min of complete ischemia and followed by 10 min reperfusion with SGLT2i, a reduction in NADH fluorescence, an increase in the NAD+/NADH ratio, a higher cytosolic calcium signal amplitude, and reduced inductivity of ventricular tachycardia were observed. These changes were not attributed to a direct inhibition of SGLT-2 [25]. In another study using constant-flow Langendorff-perfused mouse hearts, 30 min of treatment with various SGLTT-2i resulted in Na^+^/H^+^ exchanger (NHE) inhibition [26].

This suggests that SGLT-2 inhibitors can exert potential short-term effects in experimental models. However, there is a lack of studies concerning their activity in the context of AF [11], and it is not clear whether they act through protein activity modulation or by changing the expression of signaling molecules.

Our study focused on the short-term effect of SGLT-2i to evaluate the mechanisms and their potential as adjunctive therapy in paroxysmal or post-operative atrial fibrillation (POAF). Thus far, we know that in the SEARCH-AF trial, among patients with DM, SGLT-2 inhibitors—compared to other glucose-lowering therapies—led to the lowest 30-day POAF incidence [27]. However, in the overall study population, there was only a non-significant trend (*p* = 0.084) [27].

SGLT-2i reduces the risk of cardioversion and recurrences of AFwhile increasing the number of days free from AF [28,29]. A longer ischemic stroke/TIA-free time was also observed among patients with AF and DM2 [30].

SGLT-2 inhibitors are rapidly absorbed after oral administration, reaching peak plasma concentrations within 1.5 to 2.1 h [31]. If the positive short-term effects of SGLT-2 inhibitors, along with their favorable pharmacodynamics, are confirmed in AF models, this could encourage further investigation into their use as adjunctive therapy in clinical trials related to paroxysmal AF.

### 3.3. Comparison to Already Investigated Model for SGLT-2 Inhibitors Impact Mitochondrial Proteins in Atrial Fibrillation

The first study investigating the effect of SGLT-2i on mitochondrial proteins and AF inducibility was performed in rats with induced DM2 (high-fat diet/streptozotocin) after 8 weeks of treatment. The incidence of burst pacing-induced AF was 8.75% in non-diabetic rats, 85% in diabetic rats, 81.3% in rats treated with a low dose of empagliflozin, and 36.8% in those treated with a high dose [12]. This study showed that empagliflozin protected against interstitial fibrosis and the impairment of mitochondrial respiratory function, mitochondrial membrane potential, and mitochondrial biogenesis via the PGC-1α/NRF-1/Tfam signaling pathway [12]. The inducibility of AF among non-diabetic rats was very low, perhaps due to the specificity of the animal model used in this study. The preventive effect of empagliflozin was also further tested = in non-diabetic animals [12,32]; however, the hypothesis proposed by Shao et al. has not yet been verified in humans, especially in non-diabetics [12]. Additionally, studies concerning the effect of SGLT-2i on mitochondrial function in humans are limited [33].

To address this issue, we used human atrial tissue harvested from non-diabetic patients and performed a short-term experiment. Our results show an upregulation of PGC-1α in non-diabetic tissue after empagliflozin treatment under different pacing protocols. Additionally, NRF-1, a molecule downstream of PGC-1α, was upregulated during tachypacing with a higher dose of empagliflozin. However, the NRF-2 was only increased during the slow pacing with both empagliflozin concentrations.

The CaMKII-ERK1/2-Nrf2 signaling is associated with inflammation. CaMKII is considered to mediate an exercise-induced upregulation of PGC-1α [34,35]. In our study, there was a statistically significant correlation between PGC-1α and CaMKII levels during tachypacing in the control solution. However, this relationship disappeared after treatment with empagliflozin. In addition, there was a positive correlation between CaMKII and p-ERK/ERK.

### 3.4. Oxygraphy and Mitochondrial Function

In the mouse model of post-infarction heart failure, empagliflozin restored the respiratory function that was decreased by heart failure [36]. Furthermore, empagliflozin increased mitochondrial respiration with a fatty acid substrate but decreased it when it was not available. Also, muscle endurance was increased, but the peak oxygen uptake reflecting the systemic exercise capacity was not changed [37].

In another study, six weeks of treatment with SGLT2i did not affect aortic mitochondrial function in aged mice [38]. On the other hand, in 8-week-old male Sprague Dawley rats with STZ-induced DM, empagliflozin recovered impaired complex I function, improved oxidative phosphorylation, and suppressed ROS generation in atrial myocardium [39]. In 8-week-old mice with dilated cardiomyopathy induced by muscle-specific manganese superoxide dismutase deficiency (MnSOD-cKO), empagliflozin upregulated oxidative phosphorylation, increased survival, and prevented cardiac fibrosis [40]. Also, in a rat myocardial ischemia/reperfusion model, empagliflozin modulated myocardial levels of tricarboxylic acid cycle intermediates succinate, maleate, and glutamate and improved complex I-linked oxidative phosphorylation capacity and mitochondrial respiration [41].

In our study, SGLT-2i decreased the total oxygen consumption, both in the presence and absence of AF.

## 4. Materials and Methods

### 4.1. Sample Collection and Experiment Protocol

Samples of the right atrial appendage were collected from 10 patients subjected to elective cardiac surgery during the introduction of the venous return cannula to the right atrium in the initial phase of the operation and before infusion of the cardioplegic solution. All patients were hemodynamically stable in NYHA I-II stages and without overt inflammation or uncontrolled metabolic disorders. The clinical characteristics of the patients are shown in Table 1. After sample excision, it was immediately placed in the ice-cold transporting solution and transferred to the laboratory. The transporting solution consisted of a 1:1 mixture of Tyrode’s buffer with cardioplegic and cardioprotective 2,3-butanedione monoxime solution in PBS (6 g per L). Fresh tissue was rapidly embedded in 3% agar block, placed in an ice-cold oxygenated Tyrode’s buffer, and sliced into 300 μm slices using the Leica VT1200S vibratome (Wetzlar, Germany).

Then, slices were washed in ice-cold Tyrode’s buffer and placed into the 6-well cell culture plates containing one of the following solutions: Tyrode’s buffer alone (control, EMPA 0), Tyrode’s buffer with 0.2 µmol/L empagliflozin (EMPA 0.2), or Tyrode’s buffer with 1.0 µmol/L empagliflozin (EMPA 1). The plates were then covered with a lid equipped with graphite electrodes submerged in the buffer and placed on a heating pad set to 37 °C. The electrodes were connected to the stimulation output of a PowerLAB 8/30 system (AdInstruments, Dunedin, New Zealand). After a 5 min temperature equilibration period, stimulation protocols were initiated under the control of the LabCart 7 software. Three samples, each incubated in a specific solution (control, EMPA 0.2, or EMPA 1), were stimulated at a rate of 1 Hz to represent a stimulation at normal rate. Three additional samples, also incubated in the same solutions, were paced at a rate of 5 Hz to simulate tachycardia. The duration of a single stimulation impulse was set at 10 ms, with a voltage of 5 V applied in both protocols. Each stimulation protocol lasted for 30 min. After completion of the stimulation, slices were snap-frozen in liquid nitrogen and stored at −80 °C until further processing.

### 4.2. Western Blot

Frozen tissue samples were homogenized in an ice-cold lysis buffer enriched in protease inhibitor cocktail (P8340, Sigma, St. Louis, MO, USA) and phosphatase inhibitor. Protein concentration was estimated using the Bradford method. Samples containing 50 µg of protein were subjected to sulfate–polyacrylamide gel electrophoresis under reducing conditions and blotted onto nitrocellulose membranes (Bio-Rad, Hercules, CA, USA). Equal sample loading and appropriate transfer were validated by Ponceau S (P3504, Sigma, St. Louis, MO, USA) staining and further immunoblotting against GAPDH (Bio-Rad MCA 47-40).

The following primary antibodies were used: anti-OPA1 (Mitochondrial Dynamin Like GTPase, MABN737, Millipore, Burlington, MA, USA), anti-Drp 1 (Dynamin-related protein 1, ABT155, Millipore), anti-Mfn-1 (ABC41, Millipore), anti-TFAM (Mitochondrial transcription factor A, ABE483, Millipore), anti-SOD2 (Superoxide dismutase 2, PA5-30602, ThermoFisher Scientific, Waltham, MA, USA), anti-CaMKII (Ca^2+^/calmodulin-dependent protein kinase II, MA5-32125, ThermoFisher Scientific), anti-phospho-CaMKII (AB_2554441), anti-PGC-1α (Peroxisome proliferator-activated receptor gamma coactivator 1-alpha, sc-13067, Santa Cruz Biotechnology, Dallas, TX, USA), anti-NRF-1 (Nuclear respiratory factor 1, sc-33771, Santa Cruz Biotechnology) and anti-Nrf-2 (Nuclear factor erythroid 2-related factor 2, ab137550, Abcam, Cambridge, UK), anti-4-HNE (4-Hydroxynonenal, ab46545, Abcam), SIRT3 (NAD-dependent protein deacetylase sirtuin-3, sc-49744, Santa Cruz), anti-JNK1/JNK2/JNK3 (c-Jun N-terminal kinases, PA5-99529, Invitrogen, Waltham, MA, USA), anti-phospho JNK1/JNK2/JNK3 (PA5-104906, Invitrogen), anti-ERK1/ERK2 (p44/42 Mitogen-activated protein kinases, #9102, Cell Signaling, Danvers, MA, USA), and anti-phospho-ERK1/ERK2 (#9101, Cell Signaling).

Secondary antibodies conjugated with horseradish peroxidase were goat anti-rabbit HRP (Thermo Scientific 31-462) or anti-mouse HRP (Sigma A93-09). Blots were visualized using SuperSignal West Pico Plus Chemiluminescent Substrate (34580, Thermo Scientific) and exposed to the X-ray film (Carestream, Medical X-ray Blue, 771 0783 Rochester, NY, USA). Band densities were related to respective GAPDH band. To re-probe the blots, they were incubated with the Restore PLUS Western Blot Stripping Buffer (Thermo Scientific, 46430). Films were scanned and quantified using the ImageJ software, version 1.54 (National Institutes of Health, Bethesda, MD, USA).

### 4.3. Oxygraphy

The oxygraphy was performed using mitochondria extracted from atrial appendage tissue. During measurement, the adenosine diphosphate (ADP), Succinate (Succ), and Carbonyl cyanide-p-trifluoromethoxyphenylhydrazone (FCCP) were added in order to evaluate the effectiveness of respiratory chain. The groups used to divide samples were based on initial AF and SGLT-2i statues. Samples were not exposed to tachypacing. The analysis was performed using the O2ViewXP Software Version: 2.10. The protocols were based on previously published study by Frezza et al. [42].

### 4.4. Statistics

The Shapiro–Wilk test was used for the stratification of normal distribution. The parametric continuous variables were expressed by mean ± SE and analyzed using Student’s T test, and nonparametric variables were expressed as a median ± interquartile range (IQR) and analyzed using Mann–Whitney U-test. The correlation between continuous variables was analyzed using Spearman’s correlation test.

## 5. Conclusions

Empagliflozin exerts dynamic effects on the expression of PGC-1α and other proteins involved in mitochondrial function and oxidative stress in cardiomyocytes and may modulate the cellular response to tachycardia. The possible effect of SGLT-2 inhibitors should be investigated over a slightly longer time period (up to 3 h) in order to more thoroughly investigate its rapid effect on mitochondrial regulatory proteins. SGLT-2 inhibitors affect the respiratory function in AF. 

## Figures and Tables

**Table 1:** Study population characteristics.

Study Population n = 10		
General characteristics	Age (y)	62.3 ± 3.06
	Male	80% (8)
	Hypertension	90% (9)
	Dyslipidemia	80% (8)
	Ischemic Heart Disease	50% (5)
	Heart Failure	40% (4)
	Valvular Disease	80% (8)
	Mitral Regurgitation	50% (5)
	Aortic Stenosis	20% (2)
	Aortic Regurgitation	10% (1)
Treatment	ACE-I/ARB	80% (8)
	Beta-blockers	80% (8)
	MRA	40% (4)
	Diuretics	60% (6)
	Aspirin	40% (4)
	Statins	80% (8)
Echocardiographic characteristic	LVEF (%)	46.7 ± 3.07
	TAPSE (mm)	21.25 ± 3.41
	SPAP (mmHg)	32.71 ± 3.30
	LA (mm)	43.5 ± 2.78
	LVEDD (mm)	56.8 ± 3.19
	RV 4-CH (mm)	37 ± 3.21

**Table 2:** The statistically significant, strong correlations between protein expression in different settings are defined as −0.7 > R, or R > 0.7 [15].

Analyzed Relation	R	*p* Value	Analyzed Relation	R	*p* Value
EMPA 0—1 Hz			EMPA 0—5 Hz		
Drp-1 and Mfn-1	0.8285	0.04	PGC-1α and CaMKII	0.793	0.033
			CaMKII and p-ERK/ERK	0.786	0.021
EMPA 0.2—1 Hz			EMPA 0.2—5 Hz		
TFAM and SOD-2	0.9047	0.002	TFAM and p-ERK/ERK	0.90	0.037
OPA-1 and CaMKII	0.7619	0.028	NRF-2 and Drp-1	−0.886	0.019
EMPA 1—1 Hz			EMPA 1—5 Hz		
TFAM and SOD-2	0.8214	0.023	Non-statistically significant		

## Data Availability

The data used to support the findings of this study are available from the corresponding author.
